# The influence of occupational class and physical workload on working life expectancy among older employees

**DOI:** 10.5271/sjweh.3919

**Published:** 2020-12-16

**Authors:** Jolinda LD Schram, Svetlana Solovieva, Taina Leinonen, Eira Viikari-Juntura, Alex Burdorf, Suzan JW Robroek

**Affiliations:** 1 Erasmus University Medical Center, Department of Public Health, Rotterdam, The Netherlands; 2 Finnish Institute of Occupational Health, TYÖTERVEYSLAITOS, Finland

**Keywords:** Finland, occupation, older worker, physical workload factor, socioeconomic difference, work disability, working career, working years lost

## Abstract

**Objective:**

This study investigates the impact of physical workload factors and occupational class on working life expectancy (WLE) and working years lost (WYL) in a sample of older Finnish workers.

**Methods:**

A 70% random sample of Finns in 2004 was linked to a job exposure matrix for physical workload factors and register information on occupational class and labor market status until 2014. Transitions between being at work, time-restricted work disability, unemployment, economic inactivity, disability retirement, retirement and death were estimated. A multistate Cox regression model with transition-specific covariates was used to estimate the WLE and WYL at age 50 up to 63 years for each occupational class and physical workload factor for men and women (N=415 105).

**Results:**

At age 50, male and female manual workers had a WLE of 10.13 and 10.14 years, respectively. Among both genders, manual workers had one year shorter WLE at age 50 than upper non-manual employees. This difference was largely attributable to unemployment (men: 0.60, women: 0.66 years) and disability retirement (men: 0.28, women: 0.29 years). Self-employed persons had the highest WLE (11.08 years). Men and women exposed to four or five physical workload factors had about one year lower WLE than non-exposed workers. The difference was primarily attributable to ill-health-related reasons, including disability retirement (men: 0.45 years, women: 0.53 years) and time-restricted work disability (men: 0.23, women: 0.33 years).

**Conclusions:**

Manual workers and those exposed to physical workload factors had the lowest WLE. The differences in WYL between exposure groups can primarily be explained by ill-health-based exit routes.

The European ageing population is already resulting in an older workforce as well as sustainability issues of social protection systems (1). The current division of time between employment and retirement is a major challenge for policies (2). Extending working life has therefore become a strategic objective in many European countries. In order to have effective policies to extend working life, a proper understanding of labor market dynamics of years in later life spent in work, retirement or other states is needed.

Labor market dynamics in later life differ across groups of workers. Particularly workers with a low occupational class and strenuous working conditions have a higher risk to exit paid employment, often through ill-health-related exit routes (3, 4). In general, adverse working conditions are associated with premature exit from paid employment (5). Longitudinal studies from different countries reported that unfavorable working conditions such as higher physical workload increased the risk of early retirement (6–9) and disability benefits (8, 10–12). Moreover, workers with a lower occupational class are more likely to have strenuous working conditions (10, 13).

Since both a low occupational class and physically demanding work increase the risk of exit from paid employment, it is relevant to quantify their impact on cumulative measures such as working life expectancy (WLE) and working years lost (WYL). WLE expresses the number of years that persons are expected to be in paid employment until they eventually leave the labor force for retirement (14). In the past decade, several studies have estimated the expected duration of working life (14–21). So far, differences in WLE have been studied according to gender, ethnicity, socioeconomic positions, working conditions and chronic diseases. Generally, men have a higher WLE than women and persons with a high education have a higher WLE than those with a low education (14, 18, 19). The gap measure WYL reflects the working time lost due to premature exit from paid employment through various exit routes such as disability benefits, unemployment etc (14). Studies differ in how many different states for WYL are included. Some studies have studied WYL only for disability benefits (22, 23), while other studies have included other labor market states, eg, unemployment and sickness absence (20).

So far, most studies on WLE and WYL have relied on prevalence-based methods, which are unable to incorporate the complexity of laborforce dynamics with many transitions between paid employment and non-employment states (14, 24). In addition, most studies have ignored the possibility to return to paid employment after initial exit and, therefore, underestimate the WLE. Furthermore, many studies have not made the distinction between being employed and being present at work, thereby not fully capturing the WYL due to, for example, sickness absence.

In this study, a multistate Cox regression model was used to capture the dynamic patterns among transitions in and out of being at work over a ten-year period. We were, therefore, able to estimate time spent in specific exit routes including ill-health-related exit routes. The aim of this study was to estimate WLE and WYL attributable to different reasons among Finnish workers aged 50–63 years according to gender, occupational class, and physical workload factors.

## Method

### Study population

For this study, we used a 70% random sample of the working-age population from the Finnish population census taken on the 31 December 2004. Persons aged 50–63 years belonging to the workforce on 1 January 2005 were eligible to the study. Persons who did not have an occupational class or an occupational job code were excluded (about 3%). The study population consisted of 415 105 persons (204 113 men and 210 992 women), who were followed from 1 January 2005 to 31 October 2014.

### Social security system in Finland

Sickness absence is compensated after a waiting period of 10 full sickness absence days among wage earners and 1–4 days among self-employed persons, until a maximum of 300 days. Employers compensate wage earners for the waiting period. Part-time sick leave is a voluntary option for those who are incapable of performing their full duties, as determined by a physician, but who are able to work 40–60% of full time. The partial benefit is 50% of full benefit.

A disability pension can be granted to an individual whose reduced work ability due to illness is medically confirmed. To receive a full disability pension, an individual’s work ability needs to be reduced by ≥60%. A partial disability pension is granted if work ability is reduced by ≥40%. Both full and partial disability pension can be granted either on a temporary or permanent basis.

Eligibility to vocational rehabilitation from the earnings-related pension scheme is based on a medically confirmed threat of disability retirement within the next five years and on an expectation that work participation can be promoted and disability retirement postponed or prevented with vocational rehabilitation.

Unemployed job seekers receive unemployment benefits for either 300 days or 400 days depending on the length of previous employment. If the individual becomes unemployed after reaching the age of 58, the maximum is 500 days. After the basic unemployment benefit runs out, individuals can apply for labor market subsidies.

Since 2005, there has been a flexible statutory retirement age in Finland. During the time of this study, individuals could retire due to old age between the ages of 63 and 68 years. It was also possible to receive an early old-age pension at age 62. During the study period, a long-term unemployed (>500 days) person aged 60 could be granted an unemployment pension until reaching old-age retirement age. There are also special pensions for farmers.

### Labor market states

During the period 2005–2014, the registers from the Finnish Centre for Pensions provided information on earnings-related pension periods, earning periods, unemployment periods, and vocational rehabilitation periods. Information on sickness allowance periods was obtained from the register of the Social Insurance Institution of Finland (Kela).

Based on the period data on employment and benefit receipt, seven daily states were formed. Because states could take place simultaneously, we applied the following rules for state assignment:

(i) *Work*: Individuals were defined as being at work if they had an earning period and did not receive any ill-health or unemployment related benefit at the same time. There were a few exceptions; individuals on part-time sickness absence were considered to be at work, since they were required to work part-time. Individuals with a partial disability retirement could work but did not have to. If partial disability retirees had an earning period, they were considered to be at work; if not, they were coded as being on time-restricted work disability.
(ii) *Time-restricted work disability*: This state included full-time sickness absence, vocational rehabilitation due to medical reasons, temporary disability retirement and partial disability retirement (for those who did not have an earning period at the same time).
(iii) *Unemployment*: This state included any type of unemployment benefit. If a person had an unemployment and earnings period at the same time, the state of unemployment overruled. This category also included unemployment retirement.
(iv) *Economic inactivity*: This state included persons outside of the labor force for reasons other than retirement, eg, due to home care, studying or an unknown reason. This state also includes individuals who emigrated during the follow-up.
(v) *Disability retirement*: This state included only permanent full disability retirement. From this state, only transitions to old-age retirement and death were allowed.
(vi) *Retirement*: This state included old-age retirement and non-health-related types of early retirement. From this state only transitions to death were allowed.
(vii) *Death*: Based on the mortality statistics.

### Occupational class

Information on occupational class was obtained from the Finnish Longitudinal Employer-Employee Data (FLEED) of Statistics Finland. Four occupational classes were distinguished (i): upper non-manual employees, (ii) lower non-manual employees, (iii) manual workers, and (iv) self-employed.

### Physical workload

Information on occupation at baseline was also obtained from FLEED during the last week of December 2004. The occupations were classified using the Classification of Occupations 2001 by Statistics Finland, which is based on the International Standard Classification of Occupations (ISCO-88). Heavy physical work (eg, lifting and carrying heavy loads, excavating, shoveling or hammering), kneeling or squatting at work (≥1 hour a day), manual handling of heavy loads (lifting, carrying or pushing items >20 kg at least 10 times every day), working with hands above shoulder level (on average ≥1 hour per day) and awkward trunk posture (working in a forward bent posture ≥1 hour per day) were estimated with a gender-specific job exposure matrix (JEM) (25). The JEM provided the estimates for the likelihood of being exposed in the person’s occupation. Based on previous research (25), the continuous JEM values (range 0–1) were dichotomized into non-exposed (<0.40), and exposed (≥0.40 higher) workers. We calculated the total number of physical workload factors a person was exposed to and classified it into three categories: no exposure, 1–3 factors, and 4–5 factors.

### Statistical analyses

**Figure 1:**
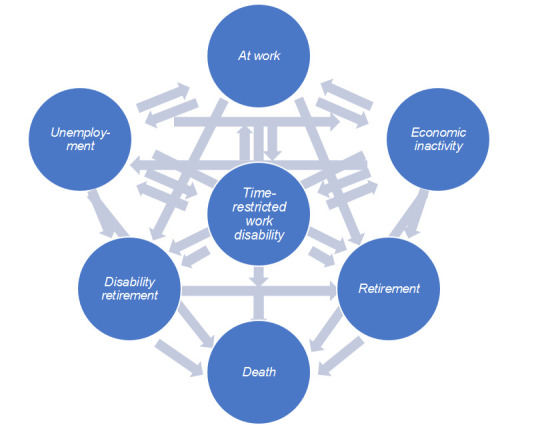
Overview of transition probabilities

*Multistate Cox regression model*. First, based on the information on daily transitions between states, transition rates were assessed in order to calculate the WLE and WYL. Individuals could move between states over time. The multistate model was composed of the previously mentioned six states and death, with death as the absorbing state. In the model, individuals with disability retirement could only move to retirement or death, and individuals in the retirement state could only move to death. A total of 27 possible transitions remained, for which a transition matrix was constructed (figure 1). Calculations were censored at 63 years, and the estimated WLE and WYL is thus based on the transitions from age 50 until 63.

Following Robroek et al (14), the R package mstate developed by Putter (26) was used to estimate cumulative transition rates and transition probabilities based on the multistate models. Analyses were performed separately for men and women. For each of the 27 transitions, occupational class and exposure category of physical workload were defined as covariates (27, 28). Using age as the underlying time axis, a Cox proportional hazards model was fitted to estimate the transition rates between states. We applied the Markov assumption that transition rates were only dependent on the current state. The baseline transition hazards were used to calculate transition probabilities for each of the possible transitions in the model. The baseline hazard was adjusted with the estimated hazard ratios (HR) by the Cox analysis.

*WLE and WYL*. The estimated transition probabilities in the multi-state model were used to calculate the expected length of stay (ELOS) in a specific state, given the current state (ELOS function in the mstate R package). We set the time horizon for the ELOS at age 63 and used the transition probabilities from the starting time (age 50 years). WLE is defined here as the number of years in the work state, conditional on being in the workforce at age 50. Bootstrapping was used to calculate the uncertainty around the expected length of stay. Bootstrapping consisted of resampling from the study population with replacement. The ELOS is calculated on the bootstrapped population, this was repeated 100 times. The lower and upper bound of the ELOS were estimated as the 2.5th and 97.5th percentile of the bootstrapped ELOS. The total WYL due to being outside of work were calculated as the difference between the potential work years until the age of 63 years (ie, 13 years) and the WLE at age 50. A sensitivity analysis for physical workload was conducted excluding the self-employed since this group had the highest WLE but also a high physical workload.

## Results

**Figure 2:**
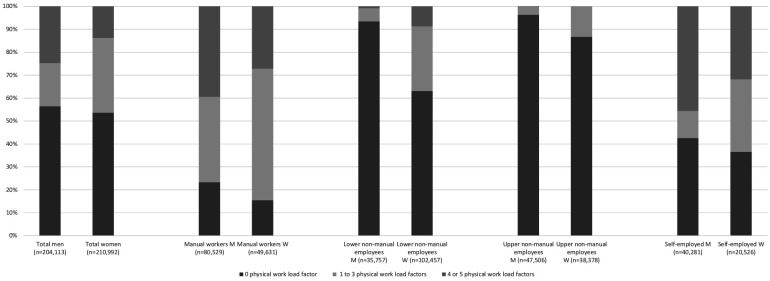
Exposure to multiple physical workload factors by gender and occupational class.

Men were more frequently exposed to physical workload factors than women, especially to manual handling of heavy loads, working with hands above shoulder level and heavy physical work (table 1). Male lower and upper non-manual employees were rarely exposed to physical workload factors, while this was more common among women in these occupational classes. Self-employed workers were often exposed to multiple physical workload factors. Overall, men were more often exposed to four or five physical workload factors than women (24.8% and 13.8%, respectively, figure 2).

**Table 1:** Proportion of persons exposed to physical workload factors, presented separately for men and women and by occupational class among Finnish workers aged 50 years and older

	Heavy physical work	Kneeling or squatting (≥1h/day)	Manual handling of heavy loads (≥20kg ≥10×/day)	Working with hands above shoulder level (≥1h/day)	Awkward trunk posture (≥1h/day)
				
	%	%	%	%	%
Total men (N=204 113)	37.5	27.4	21.9	18.7	35.1
Manual workers (N=80 529)	69.1	44.5	34.0	37.4	57.8
Lower non-manual employees (N=35 757)	1.5	1.5	0.9	2.6	5.9
Upper non-manual employees (N=47 506)	0.4	0.3	0.2	1.9	1.8
Self-employed (N=40 281)	50.1	48.3	42.3	15.3	54.9
Total women (N=210 992)	30.6	25.6	9.0	10.8	32.0
Manual workers (N=49 631)	81.4	42.7	6.7	28.6	66.5
Lower non-manual employees (N=102 457)	15.2	23.7	8.8	3.6	20.2
Upper non-manual employees (N=38 378)	0.1	2.9	0.0	5.6	7.6
Self-employed (N=20 526)	41.9	35.6	32.4	13.5	53.0

Table 2 shows the WLE at age 50 and WYL for each occupational class, separately for men and women. The WLE for men and women was 10.13 and 10.14 years, respectively. For both genders: (i) the WLE was around 10.50 years among upper level employees and 9.51 years among manual workers, with a gap of one year between these occupational classes; (ii) the largest WLE was found for the self-employed, slightly above 11 years; (iii) a total of 3.49 years of being at work was lost, of which 44% was due to ill-health-related reasons (time-restricted work disability, disability retirement, or death).

**Table 2:** Working life expectancy (WLE) and working years lost (WYL) among Finnish workers aged 50–63 years by gender and occupational class [CI=confidence interval.]

	WLE	WYL due to					
	
		Time-restricted work disability	Disability retirement	Death	Unemployment	Economic inactivity	Retirement
						
	WLE (95% CI)	WYL (95% CI)	WYL (95% CI)	WYL (95% CI)	WYL (95% CI)	WYL (95% CI)	WYL (95% CI)
Men	10.13 (10.11–10.15)	0.41 (0.41–0.42)	0.66 (0.64–0.67)	0.20 (0.20–0.21)	0.64 (0.64–0.65)	0.41 (0.41–0.42)	0.54 (0.53–0.55)
Manual workers	9.51 (9.48–9.54)	0.48 (0.47–0.48)	0.83 (0.81–0.85)	0.23 (0.22–0.24)	1.01 (0.99–1.02)	0.40 (0.39–0.41)	0.55 (0.54–0.56)
Lower non-manual employees	10.08 (10.06–10.10)	0.43 (0.42–0.43)	0.68 (0.66–0.69)	0.21 (0.20–0.21)	0.64 (0.63–0.65)	0.42 (0.41–0.43)	0.55 (0.54–0.56)
Upper non-manual employees	10.50 (10.48–10.52)	0.38 (0.37–0.39)	0.55 (0.53–0.56)	0.19 (0.18–0.20)	0.41 (0.40–0.42)	0.43 (0.42–0.44)	0.54 (0.54–0.55)
Self-employed	11.08 (11.06–11.11)	0.29 (0.29–0.30)	0.37 (0.35–0.38)	0.17 (0.16–0.18)	0.17 (0.16–0.17)	0.41 (0.40–0.42)	0.52 (0.51–0.52)
Women	10.14 (10.12–10.16)	0.55 (0.54–0.55)	0.61 (0.60–0.63)	0.09 (0.09–0.10)	0.62 (0.61–0.62)	0.33 (0.33–0.34)	0.66 (0.65–0.67)
Manual workers	9.51 (9.47–9.54)	0.65 (0.64–0.66)	0.79 (0.76–0.81)	0.10 (0.09–0.11)	1.03 (1.01–1.05)	0.27 (0.27–0.28)	0.66 (0.65–0.66)
Lower non-manual employees	10.12 (10.09–10.13)	0.56 (0.56–0.57)	0.63 (0.61–0.65)	0.09 (0.09–0.10)	0.61 (0.60–0.62)	0.32 (0.32–0.33)	0.67 (0.66–0.67)
Upper non-manual employees	10.53 (10.51–10.55)	0.48 (0.47–0.49)	0.50 (0.49–0.52)	0.09 (0.09–0.09)	0.37 (0.36–0.38)	0.36 (0.36–0.37)	0.66 (0.65–0.67)
Self-employed	11.05 (11.02–11.09)	0.33 (0.32–0.34)	0.33 (0.31–0.35)	0.09 (0.08–0.10)	0.15 (0.14–0.16)	0.42 (0.40–0.44)	0.63 (0.62–0.64)

There were small differences in WYL between men and women. Women lost slightly more years due to time-restricted work disability and retirement, and men lost slightly more years due to disability retirement and death. Absolute occupational class differences in WYL between upper non-manual employees and manual workers were largest for unemployment (men: 0.60, women: 0.66 years) and disability retirement (men: 0.28, women: 0.29 years).

**Figure 3:**
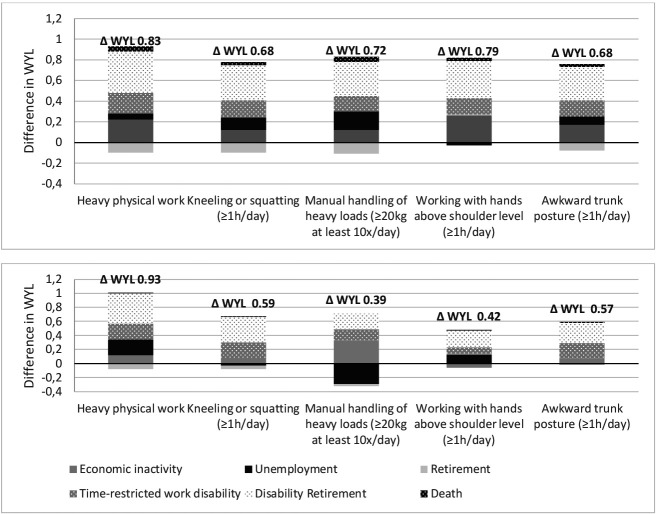
Difference in working years lost (Δ WYL) from 50 to 63 years by gender between persons exposed to specific physical workload factors and persons not exposed to these specific physical workload factors (men in upper panel, women in lower panel).

Table 3 shows WLE and WYL for the different groups by the number of physical workload factors, separately for men and women. The difference in WLE between persons not exposed and persons exposed to four or five physical workload factors was 0.90 years for men and 0.98 years for women. Compared to persons with no exposure to physical workload factors, persons with exposure to 4 or 5 physical workload factors lost most years due to disability retirement (men: 0.45, women: 0.53 years) and time-restricted work disability (men: 0.23, women: 0.33 years). When analyzing each physical workload factor separately, the largest difference in WYL between exposed and non-exposed workers was for physical heaviness (men: 0.83 years, women 0.93 years) (figure 3). For each factor of physical workload, time-restricted work disability and disability retirement had the largest contributions to the WYL.

**Table 3:** Working life expectancy (WLE) and working years lost (WYL) among Finnish workers aged 50–63 years by gender and exposure to multiple physical workload factors. [CI=confidence interval.]

Physical workload factors	WLE	WYL due to					
	
		Time-restricted work disability	Disability retirement	Death	Unemployment	Economic inactivity	Retirement
						
	WLE (95% CI)	WYL (95% CI)	WYL (95% CI)	WYL (95% CI)	WYL (95% CI)	WYL (95% CI)	WYL (95% CI)
Men							
0	10.44 (10.43–10.46)	0.33 (0.33–0.34)	0.50 (0.49–0.52)	0.19 (0.18–0.19)	0.58 (0.57–0.59)	0.37 (0.37–0.38)	0.58 (0.57–0.59)
1–3	10.03 (10.01–10.05)	0.43 (0.43–0.44)	0.70 (0.68–0.71)	0.21 (0.20–0.22)	0.67 (0.67–0.68)	0.43 (0.42–0.44)	0.53 (0.52–0.53)
4–5	9.54 (9.50–9.58)	0.56 (0.55–0.57)	0.95 (0.93–0.98)	0.23 (0.22–0.24)	0.76 (0.75–0.78)	0.48 (0.47–0.49)	0.47 (0.47–0.48)
Women							
0	10.42 (10.40–10.44)	0.45 (0.45–0.46)	0.47 (0.45–0.48)	0.09 (0.09–0.10)	0.61 (0.60–0.62)	0.29 (0.28–0.30)	0.67 (0.67–0.68)
1–3	9.98 (9.95–10.00)	0.60 (0.59–0.61)	0.69 (0.67–0.71)	0.10 (0.09–0.10)	0.63 (0.62–0.64)	0.36 (0.35–0.36)	0.65 (0.65–0.66)
4–5	9.44 (9.41–9.48)	0.78 (0.76–0.79)	1.00 (0.96–1.03)	0.10 (0.09–0.11)	0.63 (0.61–0.65)	0.42 (0.40–0.43)	0.63 (0.62–0.64)

The analysis of the influence of physical workload on WLE within each occupational class showed consistent differences of 0.48–0.72 years between exposure to four or five physical workload factors versus no exposure (table 4). When comparing the most advantaged group (upper non-manual employees with 0 exposures) and the most disadvantaged group (manual workers with four or five exposures), the difference in WLE was 1.56 for men and 1.59 for women. The WYL were attributable primarily to disability retirement in both genders, but also unemployment (both genders) and time-restricted work disability (women).

**Table 4:** Working life expectancy (WLE) and working years lost (WYL) among Finnish workers aged 50–63 years by gender, occupational class and exposure to multiple physical workload factors.

Occupational class	Number of exposures	WLE	WLE due to					

			Time-restricted work disability	Disability retirement	Death	Unemployment	Economic inactivity	Retirement
Male								
Manual	0	9.83	0.39	0.65	0.21	0.98	0.36	0.59
Manual	1–3	9.49	0.49	0.85	0.23	1.01	0.40	0.54
Manual	4–5	9.11	0.61	1.11	0.25	1.01	0.44	0.48
Lower non-manual	0	10.31	0.35	0.54	0.19	0.64	0.39	0.59
Lower non-manual	1–3	10.01	0.45	0.71	0.21	0.65	0.44	0.53
Lower non-manual	4–5	9.66	0.56	0.93	0.23	0.66	0.48	0.49
Higher non-manual	0	10.67	0.32	0.44	0.18	0.41	0.41	0.57
Higher non-manual	1–3	10.40	0.41	0.59	0.20	0.42	0.46	0.53
igher non-manual	4–5	10.08	0.51	0.79	0.21	0.42	0.51	0.48
Self-employed	0	11.16	0.25	0.31	0.16	0.17	0.41	0.54
Self-employed	1–3	10.94	0.33	0.42	0.18	0.17	0.46	0.50
Self-employed	4–5	10.68	0.42	0.56	0.19	0.17	0.51	0.46
Female								
Manual	0	9.67	0.53	0.58	0.09	1.22	0.24	0.67
Manual	1–3	9.45	0.68	0.82	0.10	1.02	0.28	0.65
Manual	4–5	9.11	0.86	1.14	0.10	0.85	0.31	0.63
Lower non-manual	0	10.32	0.47	0.49	0.09	0.66	0.29	0.68
Lower non-manual	1–3	10.03	0.61	0.69	0.10	0.57	0.34	0.66
Lower non-manual	4–5	9.64	0.77	0.97	0.10	0.49	0.38	0.64
Higher non-manual	0	10.70	0.42	0.41	0.09	0.38	0.34	0.67
Higher non-manual	1–3	10.39	0.53	0.58	0.09	0.34	0.40	0.66
Higher non-manual	4–5 a	·	·	·	·	·	·	·
Self-employed	0	11.09	0.31	0.30	0.09	0.15	0.43	0.63
Self-employed	1–3	10.80	0.40	0.43	0.09	0.15	0.51	0.62
Self-employed	4–5	10.46	0.51	0.60	0.10	0.14	0.58	0.61

a Number of females in higher non-manual occupational class with exposure to 4 or 5 physical workload factors is too low to estimate WLE and WYL.

The sensitivity analysis, excluding the self-employed, showed a larger difference than the main analysis in WLE between exposed and non-exposed workers (supplementary table S1, www.sjweh.fi/show_ abstract.php?abstract_id=3919). Workers who were not exposed to physical workload factors had 1.34 years (men) and 1.04 years (women) higher WLE than those exposed to four or five factors. Absolute differences in WYL between the groups were largest for disability retirement (men: 0.61, women: 0.59 years), followed by unemployment for men (0.43 years), and time-restricted work disability for women (0.37 years).

## Discussion

This study utilized longitudinal register data from 2005 to 2014 to estimate WLE at age 50 in the Finnish workforce overall as well as by occupational class and exposure to physical workload factors. Overall, the estimated WLE at age 50 was 10.1 years with no gender difference. Both male and female upper non-manual employees had a one year longer WLE at age 50 than manual workers, and self-employed workers had the highest WLE. Workers exposed to four or five physical workload factors also had a lower WLE than non-exposed workers, with a larger difference among women (0.98 years) compared to men (0.90 years). Differences in WYL between manual workers and upper non-manual employees were largest for unemployment (men: 0.60 years, women: 0.66 years). The difference between workers with exposure to multiple physical workload factors and non-exposed workers was largest for disability retirement (men: 0.45 years, women: 0.53 years).

Some previous studies have examined socioeconomic differences in WLE or WYL in Finland (15, 19, 22), and other countries (14, 20, 21). The absolute values reported in the studies are not directly comparable due to differences in definitions of working life, study populations, study periods, statutory retirement ages, and estimation methods. For instance, WLE has previously been based on time spent in paid employment instead of time actually being present at work (eg, 14, 18.). This is an important distinction as the latter excludes employment periods during which a person is on sick leave. Furthermore, the definition of being at work may vary depending on whether time-restricted work disability with part-time employment is defined as work or work-related disability. Likewise, crucial assumptions pertain to selection and definition of included states. Nevertheless, the findings of the current study are in line with previous studies with regard to the lowest WLE in most disadvantaged groups, such as among persons with a low education, manual workers and those with physically demanding work (eg, 14, 15, 19–22.). These findings ask for prevention of premature exit from paid employment due to ill health among older workers with physically demanding work.

This study showed differences in WLE across occupational classes among both men and women. Manual workers had a WLE of 9.5 years at the age of 50, while upper non-manual employees had a WLE of 10.5 years, resulting in a one year difference. In a previous study on WLE in Finland, using the classical Sullivan method on repeated cross-sectional information from 1989–2012, much larger differences between upper non-manual and manual employees were reported throughout the study period, with differences of 3.65 years for men and 3.63 years for women in the last year (15). A primary reason for the difference in the results between the above mentioned study and the current one is likely the selection of the study population. The current study excluded those who were outside the workforce at baseline, resulting in a healthier study population particularly in physically demanding occupations. Additionally, Leinonen et al (15) examined employment participation until the end of working careers, ie, beyond the limit of age 63, as is used in the current study. Another study from the authors indicated that manual workers quit paid employment more often at the first possible age of retirement, whereas upper non-manual employees extended their working lives to an older age (29). Finally, Leinonen and colleagues measured labor market status only at the end of each year (15).

No gender differences were seen in WLE. The current study had a selected study population, ie, those who were in the workforce at age 50 because our focus was on the effect of physical work exposures. This may explain why no gender differences were found in contrast to previous Finnish findings, which showed longer WLE at age 50 among women compared with men (15, 16).

Traditional research in occupational health focuses on questions such as “what is the increased risk of workers with a high physical workload to exit paid employment?” (11) or “to what extent does physical workload explain occupational class inequalities in disability retirement for older workers?” (30). Our study adds a cumulative measure over the remaining working life course to the previous body of knowledge. WYL due to a range of states are calculated for different occupational classes as well as for different exposures to physical workload factors. In line with our results, other studies have reported disability retirement as an important reason for WYL (14–16, 21, 22).

Perhaps a counterintuitive conclusion based on the presented figures was that self-employed persons had the highest WLE, despite high levels of physical workload. Self-employed persons may be among men eg, freelance construction workers or farmers and among women service workers. A previous study from Finland reported that entrepreneurs (or freelancers) had a slightly lower WLE at age 50 compared to the upper non-manual class (15). Another Finnish study on pensions and pensions earnings found that self-employed workers have the longest working careers in Finland when taking into account their total working life (31). Reasons for a high WLE among self-employed seem to be twofold. Part of the self-employed enjoy working and continue until an older age, whereas another part have not contributed sufficiently to their pension insurance and need to continue working to be able to receive sufficient pension (32). Studies in The Netherlands showed that financial stimulants and autonomy at work played a role in prolonging working lives (33).

### Strengths and limitations

Firstly, one of the main strengths of our study is the use of longitudinal data from a representative national sample of 70% of the population and data on episodes of employment and benefit receipt derived from complete national registers. Secondly, the national registers were used to distinguish between multiple detailed labor market states, while other WLE studies have had to rely on more limited data such as main source of income (14) or self-reported employment state (18), which limits the amount and the precision of distinguishable labor market states. Thirdly, information on physical workload factors has been derived from JEM by linking to job titles. However, using the JEM with the national representative sample did mean we lost some observations due to missing job codes. Fourthly, with the use of a multistate Cox regression model the possibility to re-enter into the labor market was taken into account.

The study also has some limitations. Firstly, the division into labor market states is somewhat arbitrary since, in the model, persons cannot be in multiple labor market states at one time – although in practice this can be the case. Priority was given to non-working states in order to minimize underestimation of WYL. For example, work done during unemployment or full retirement is often very minimal. As an exception, work done during partial work disability was categorized as work, since it was expected to involve a larger amount of working time. Part-time sickness beneficiaries are always required to work 40–60% of normal working time and partial disability pensioners can receive up to 60% of the previous earning level without losing the benefit. Secondly, occupational titles were only measured at the end of 2004, and workers could have changed jobs in the following years. However, since the study population was aged 50–63 years in 2005, it can be hypothesized that major job changes were not likely. Thirdly, although we consider the JEM to define the exposure to physical workload factors as a strength, the JEM is developed in a broader age group (18–64 years) compared to our study sample (50–63 years). It might be that the true exposure in this older age group differs from the exposure to physical workload factors in the broader age group that was used to develop the JEM. In addition, the group-based assessment of physical workload factors in the JEM may differ from individual-based assessment of physical workload factors, but we lack information to hypothesize whether this would result in an underestimation or overestimation of differences in WLE between exposed and non-exposed. Fourthly, during the follow-up period, there were several changes in the economic situation and in sickness absence legislation. Differences over time, as a result of economic and legal changes in WLE and WYL due to physical workload factors, were not part of the current study and would be of interest for future research. Lastly, the absolute values of our study are not easily generalized to other populations. While WYL due to high physical load and lower WLE in lower occupational classes have been reported before, their exact magnitude may vary across populations with different arrangements for disability and retirement benefits.

### Concluding remarks

This study shows differences in WLE by occupational class and number of physical workload factors in later working life among both men and women. Both manual workers and workers with exposure to multiple physical workload factors have a reduced WLE. The difference in WLE between occupational classes can be primarily explained by WYL due to unemployment and, to a smaller extent, ill-health. The difference between the exposure groups is primarily attributable to WYL due to ill-health-related reasons, including disability retirement and time-restricted work disability.

## Funding

This work was conceived with financial support from ZonMW (grant no. 208060001) within the Joint Programming Initiative More Years Better Lives (WORKLONG project) framework. Additional financial support was received through The Finnish Work Environment Fund (3507301) Strategic Research Council at the Academy of Finland (SRC-STN 303534), NordForsk (76659), Nordic Council of Ministers (NCM 101250) and the European Cost Action OmegaNet through a short-term scientific mission (ECOST-STSM-Request- CA16216-45067).

## Supplementary material

Supplementary material
