# Supplementary material for: The influence of occupational class and physical workload on working life expectancy among older employees
Source: Scand J Work Environ Health. 2020 Dec 16;47(1):5–14. doi: 10.5271/sjweh.3919 (PMC7801139; doi:10.5271/sjweh.3919)
Supplement: Supplementary material [file SJWEH-47-5-S001.pdf]

The influence of occupational class and physical workload on working life expectancy among older employees [1]

by Jolinda LD Schram, MSc, Svetlana Solovieva, PhD, Taina Leinonen, PhD, Eira Viikari-Juntura, PhD, Alex Burdorf, PhD, Suzan JW Robroek, PhD [2]

1. *Supplementary material*
2. *Correspondence to: Suzan J W Robroek, Department of Public Health, Erasmus MC, Rotterdam 3000 CA, The Netherlands. [E-mail: s.robroek@erasmusmc.nl]*

Supplementary Table S1 – Working life expectancy (WLE) and working years lost (WYL) among Finnish workers aged 50-63 years by gender and exposure to multiple physical workload factors (without self-employed)

		WYL due to					
	WLE	Time-restricted work disability	Disability Retirement	Death	Unemployment	Economic inactivity	Retirement
Men							
0 poor working conditions	10.39	0.33	0.50	0.18	0.63	0.37	0.60
1-3 poor working conditions	9.79	0.45	0.75	0.21	0.83	0.40	0.57
4-5 poor working conditions	9.05	0.59	1.11	0.25	1.06	0.41	0.53
Women							
0 poor working conditions	10.37	0.46	0.47	0.09	0.62	0.29	0.69
1-3 poor working conditions	9.91	0.62	0.71	0.10	0.68	0.28	0.70
4-5 poor working conditions	9.33	0.83	1.06	0.10	0.72	0.26	0.70
